# Business Simulation Games Analysis Supported by Human-Computer Interfaces: A Systematic Review

**DOI:** 10.3390/s21144810

**Published:** 2021-07-14

**Authors:** Cleiton Pons Ferreira, Carina Soledad González-González, Diana Francisca Adamatti

**Affiliations:** 1 Research and Innovation Department, Instituto Federal de Educação, Ciência e Tecnologia do Rio Grande do Sul, Rio Grande 96201-460, Brazil; 2 Computer Engineering and Systems Department, Universidad de La Laguna, Avda. Astrofísico F. Sanchez s/n, 38204 La Laguna, Tenerife, Spain; carina.gonzalez@ull.edu.es; 3 Centro de Ciências Computacionais, Universidade Federal do Rio Grande, Av. Itália, s/n, km 8-Carreiros, Rio Grande 96203-900, Brazil; dianaadamatti@furg.br

**Keywords:** business simulation game, serious game, EEG, eye tracking, learning, neuroscience

## Abstract

This article performs a Systematic Review of studies to answer the question: What are the researches related to the learning process with (Serious) Business Games using data collection techniques with Electroencephalogram or Eye tracking signals? The PRISMA declaration method was used to guide the search and inclusion of works related to the elaboration of this study. The 19 references resulting from the critical evaluation initially point to a gap in investigations into using these devices to monitor serious games for learning in organizational environments. An approximation with equivalent sensing studies in serious games for the contribution of skills and competencies indicates that continuous monitoring measures, such as mental state and eye fixation, proved to identify the players’ attention levels effectively. Also, these studies showed effectiveness in the flow at different moments of the task, motivating and justifying the replication of these studies as a source of insights for the optimized design of business learning tools. This study is the first systematic review and consolidates the existing literature on user experience analysis of business simulation games supported by human-computer interfaces.

## 1. Introduction

The search for the best academic training of its students, increasingly aligned with the needs of organizations, has led educational institutions to use support tools in the development and improvement of knowledge, skills, and competencies [1]. Therefore, technology in education is becoming increasingly relevant, and many institutions have been increasing their virtual education strategies due to the COVID-19 pandemic [2]. Thus, the importance of a deeper scientific look at the possible contributions of the Business Simulation Games (BSG) emerges [3,4].

The few types of research related to the design of BSG, mainly associated with the user experience, point out the need for contributions from other studies. In this sense, the approximation results from investigations with Serious Games can provide relevant insights into the theme. They are active learning tools that present similar didactic principles of demonstrative, activity, accessibility, a combination of theory and practice, scientific character, and involvement, to develop skills and knowledge for its users [5]. With this perspective of analysis, researches with serious games, and applicable to BSG show that to meet the learning demands, it is necessary to contribute beyond the resources of immersion, interactivity, engagement, and similarity to the real world [2,6]. Also, it is needed to include different aspects such as proposed objectives, practical challenges, and ongoing feedback on the learner’s performance, which makes evaluation difficult since there is no predetermined object. Still, a space for experimentation with the possibility of successes and errors, in which it is essential to monitor and evaluate the entire process beyond a simple result [7]. In addition to these issues, there are few investigations on the effective contributions of these tools to socioemotional aspects and related to the cognition of their users [8,9].

The development of Serious Games must involve a team integrating people from different areas and knowledge so that each one with their expertise can contribute with elements to a final product that offers its full potential to users [6]. From another perspective, Serious Games can also benefit from information from physiological measurement devices that, in an integrated way, analyze the user experience and can contribute to their design process [10,11,12].

In line with these emerging issues, the proposal of this work is presenting a multidisciplinary study pointing out the current stage in literature areas of Business/Serious Game, Education, and Neuroscience from the following question:

*What are the studies related to the learning process with Business (Serious) Games using data collection techniques with electroencephalogram signals or eye tracking*?

Although the use of sensors to obtain feedback on human behavior in specific tasks is a widely explored scientific field, especially in the health area, when the purpose is to study their possible contributions to digital business learning tools in teaching spaces, the limitation of investigations is evident. As a marketing strategy for schools and universities, its use is pointed out as a differential in preparing the future professional, but what is effectively known about its benefits is the result of qualitative research. The motivation for this work, while making it unique, arises to bring together the existing findings on ET and EEG devices as a resource for analyzing elements in games and simulators for the development of equivalent studies with BSG. Other technologies can also provide insights, including those mentioned in the studies considered by this SR. Still, the option to focus the scope on these two interfaces, both commercially and scientifically consolidated, is to provide quantitative investigations with BSG in the short term.

The records found in response to this question address the monitoring of physiological characteristics during the user experience of different serious games. It is intended to contribute to the design and improvement of virtual environments for business learning.

## 2. Background

### 2.1. Approaching Serious Games and Neuroscience for a Better Learning

The development and search for a continuous improvement of skills and competencies in line with current personal and professional demands, which go beyond technical knowledge, has been recommended by organizations recognized worldwide. And their research points to the need to constantly improve high-level thinking, creativity, collaboration, and the ability to analyze problems and make decisions, among other elements, as the secret to success in life [9,13,14].

One of the biggest challenges of teaching has been establishing the balance between theory and practice. Commonly, the knowledge developed in the academic environment presents a minimal view of organizations and their processes, as a static model, without the possibility of changes [15]. Despite some research showing that only a tiny part of the knowledge acquired by the students can be applied in professional life [16,17], this scenario has been changing. A recent survey of higher education students shows the recognition of digital media as a way of empowerment that allows them to get the most out of educational opportunities because they are more flexible and adaptive. Also, this study highlights that it is a way to develop digital skills and transversal skills in all contexts of life, whether personal, social, or professional. In this sense, it is already a reality that institutions worldwide, aware of the potential of this digital environment, are adopting Serious Games, including business ones, as a learning strategy [18,19]. In these virtual tools, the learner can experience dynamic situations in the most diverse environments, approaching different realities of the organization, building new possibilities of organizational processes. The student interacts as a key figure so that the exercise of planning, organization, and decision-making is as or more important than the final result [20]. Added to the impact of serious games, the development of skills such as analytical thinking, transfer and retention of knowledge, motivation, adaptive learning, change in the way of seeing and facing situations [21,22,23], it can contribute to obtaining and redeeming content, offering subsidies for a possible reconstruction of a moment or context.

From a neuroscientific point of view, when dealing with something unusual and different, the brain tries to connect to an existing neural network providing new information. This information potentially increasing the retention of information, considering that a new situation makes sense or has real meaning when it fits into a pre-existing neuronal pattern. [8,24,25].

Another relevant element of behavior is a close correlation between emotion and brain functions. Specifically, the limbic and paralimbic systems, the vegetative nervous system, and reticular activation are closely related to the processing and control of emotional behavior [26]. As for affectivity, the serious game contributes to the development of technical and intellectual knowledge, obtained through a constant integration of the functional areas of the brain, improving learning. Concerning group learning, the contribution through serious games is given by the dynamics that explore teamwork, which brings a more independent learning character and helps develop social and interpersonal skills. [21,27,28].

Studies in the field of neuroscience have offered insights into simulation and Serious Games. They have collaborated with elements to create advanced learning models that increase brain volume, develop neuronal plasticity from its use, and improve visual acuity, increasing motor coordination and memory beyond simple recognition. [29,30,31].

### 2.2. Definition and Elements of Business Simulation Games (BSG)

Simulators and Serious Games are digital environments designed with the objective of teaching or training through an experience that goes beyond entertainment and fun (without necessarily excluding such characteristics), using technological resources, and following gamification methods in an approach to daily situations. According to [32], “*A simulation is a working representation of reality; it may be an abstracted, simplified or accelerated model of a process. It purports to have a relevant behavioral similarity to the original system*”. The same author highlights that simulation and serious games “*combines the features of a game (competition, cooperation, rules, participants, roles) with those of a simulation*,” and further concludes that “A serious game is a simulation game if its rules refer to an empirical model of reality”.

The BSG constitutes a real example of an e-learning methodology in business education [33,34,35], due to it manages to bring, to the virtual format, real aspects of a business environment, enabling apprentices to manage companies in risk-free scenarios and offering a broad view of the strategic functions that permeate corporations, in an attractive and interactive approach [36]. Any game that presents an organizational setting and incorporates whatever the characteristics of the “business world” should be considered a BSG and should be categorized as a “simulation game” or “serious game,” unless it offers an educational approach wrong or that manifest deliberately unrealistic reactions to the choices of its users [37]. A BSG can be used as a learning tool, simulating market trends or corporate behaviors to provide a strategic view [38,39,40], be it a new business or already established.

The first and more recognized classification developed for the BSG [38] considered that this instrument under the design aspect could present itself as: total enterprise or functional interacting or noninteracting, and computer or noncomputer; and according to their expected use: as a part of a general management training program; for selling new techniques or procedures, or for conducting research. As a method that presents itself to conduct more effective learning, simulation games should provide and improve skills and instigate the evaluation of results and feedback for those who use them [41]. It means adding features related to the fidelity, verification, and validation of the proposed model and the tool itself, such as interactivity and immersion capacity [42], such as the potential to establish the sequence in decisions, in addition to an interface and friendly appearance [37].

Regarding the systematization of research on the modeling of a game, the Taxonomy of Computer Simulations [43] and then adapted for the BSG [37] considers the following macro-categories: Environment of application; Design elements of the user interface; Target groups, Goals & Feedback; User relation characteristics; Characteristics of the simulation model.

Interactive systems design must contemplate several aspects, such as attention, essential human capacity in executing tasks. The author highlights the importance of working on resources that recognize recourse and devices such as assistants and automatic error checkers for possible deviations in attention in interactive systems design. These resources are significant to simulate the reasoning of an expert professional in a specific area of knowledge, capable of offering suggestions and advice to its users [42]. The author also highlights the relevant aspect of social interaction in this context, as individuals’ thoughts, feelings, and behaviors are influenced by the presence of others. They do not exist; they decide that a simulation system’s social effect is significant for its users.

It is essential to consider the possibility of investigating new methodologies and devices to monitor and analyze the user experience of BSG, considering the high level of complexity and multiplicity of operational and project requirements presented it can contribute with essential elements to guide its design and success as a learning tool.

### 2.3. Human-Computer Interfaces Supporting the BSGs User Experience

Physiological and neuroscientific techniques support the development of human-computer interfaces with investigations from psychophysics, cognitive neuroscience, and computer science. Together have strived to understand the interaction of people with technologies, including learning through games. A recent scientific publication presented the most prominent methods in this area [44], highlighting electroencephalography (EEG) and eye tracking (ET). It considered the two support techniques most used and more effective in the business context [45,46,47,48]. It presented its forms of operation, functionalities, limitations, and possible contributions in the context of the BSG.

#### 2.3.1. Electroencephalography (EEG)

Electroencephalography, based on the capture of brain signals, has been one of the most used non-invasive methods to capture and analyze human behavior. To record these signals, the person studied wears a cap with electrodes attached to it, establishing contact with the brain. The equipment captures minor variations in its activity from stimuli during a predefined activity. At each moment when the neuron is activated, a polarization occurs, and a consequent action potential is transmitted to other neurons, thus creating a current of information that generates an electrical activity captured by the electrodes and, subsequently, is sent to the module. Electronic for a process of filtering and subsequent data mining [49]. The signals captured through the electrodes reflect the intensity of the brain waves generated in the scalp. The most advanced EEG devices have a high temporal resolution, capturing activity in milliseconds, and excellent precision in spatial resolution. The equipment is currently being made available in portable sizes and has relatively affordable prices in some versions, allowing a greater diffusion in its use [50].

The application of EEG for the recognition of emotion and mental effort has already been the objective of a reasonable number of studies [51,52,53,54], using different methodologies for collecting, classifying, and analyzing signals for applications such as medicine and education. Although the temporal resolution of most EEG devices is considered high, as already mentioned, the spatial resolution is deemed to be limited. However, it is still possible to identify the general origin of the EEG, providing important information about the types of mental processes occurring in a given moment or situation, which can already be considered relevant to understanding human behavior. [44]. In necessary research [55], several models for data extraction with EEG in human activities methods were analyzed and proposed from collected data. In other investigations, measurements of EEG signals were applied in the analysis of the response of game users [56,57], and expert researchers discussed methodological advances in player experience and playability, highlighting EEG-based results as a good measure for analyzing cognitive behavior [58].

The experience with the BSG provides various mental and emotional states in the definition of strategies and decision-making when playing. Thus, the EEG proves to be a very recommendable monitoring sensor. It allows the identification of the activation of different brain regions, such as the frontal lobe and memory-related areas, happening in situations requiring figurative and analytical reasoning, and occipital and parietal lobe areas, during movement perception and demand for visuospatial attention [56,57,58].

#### 2.3.2. Eye Tracking (ET)

Eye tracking is a method that registers visual attention directly and continuously. This method has been applied generally to monitor the user’s attention, using a device that emits infrared rays directly into their eyes, making it possible to determine with considerable precision where you are looking. Also, it allows measuring eye movements and data related to fixations, visions, and regressions [59]. ET devices have been applied in the diagnostic area through eye movement records and corresponding visual behavior, providing results very quickly [60,61]. The following metrics can be collected through ET during system evaluation of a predefined activity, including performance measures: Efficiency and Effectiveness; for process measures: Number of fixations and Fixations, Attentional switching, and scan path similarity [62,63].

The use of ET to monitor visual behavior in a given situation provides patterns that reflect the most varied interactions between the stimulus received, the region of eye activation, the neuron temporal response characteristics, and the positions of the retina along with the image movements.

The wide variation in activity patterns suggests that, during the visualization of a stationary scene, some cortical neurons transmit information about the occurrences and directions of the balconies. In contrast, others assume the role of encoding details of the retinal image, playing a double benefit: monitoring brain activity and acting on oculomotor function [62]. Considering the reported functionalities, currently, the ET devices have been used in fields such as marketing [47,48,64], analysis of the usability of games and virtual environments [65,66,67], and recently in human behavior and applied neuroscience research [62,68]. Also, it includes the use of EEG- and ET-based measurement interfaces in an integrated manner, comparing and complementing results [65].

The structure of a BSG usually must establish a series of managerial information that require the player’s attention, and at the same time, provide enough elements for their success in the experience with the tool. The sensing of these behaviors through ET allows us to understand how the player takes advantage, or not, of the potential offered by the game from the control of the saccades and eye fixations. Other features such as heat maps identify levels of more or less involvement in certain screens in analyzing what sustains attention and motivation.

## 3. Materials and Methods

### 3.1. Search Strategy and Selection Criteria

This Systematic Review (SR) was performed using the PRISMA 2020 statement [69] as a guideline methodology. The Eligibility Criteria considered for this SR initially should answer the question: What is the research related to the learning process with Business (Serious) Games using data collection techniques with electroencephalogram signals (EEG) and/or eye tracking (ET)? According to inclusion and exclusion criteria. The main Inclusion criteria were the publications that are thematically linked with the keywords: business game (BG), serious game (SG), electroencephalography (EEG), eye tracking (ET), learning (LRN) and neuroscience (NSC), and for the exclusion criteria, articles not written in English, Portuguese or Spanish language.

The information sources included multiple scientific databases and registers, considering scientific papers available in open access or with access provided by Universidad de La Laguna and Universidade Federal do Rio Grande. Articles collected for this research were founded by searching through the following bases: IEEE, PubMed, Scopus, Springer, and Web of Science. All databases were accessed between 26 April and 7 May 2021.

The search strategy for publications was performed using filters that assure Eligibility criteria explained by PRISMA item 5.

A first combination with the following strings “Business Game” OR “Serious Game” with “EEG,” “ET,” “Learning,” “Neuroscience” returned the following results:

(1) “Business Game” AND “EEG” AND “ET” AND “Learning” AND “Neuroscience” = 0 results;
(2) “Business Game” AND “EEG” AND “Learning” AND “Neuroscience” = 0 results;
(3) “Business Game” AND “ET” AND “Learning” AND “Neuroscience” = 0 results;
(4) “Serious Game” AND “EEG” AND “ET” AND “Learning” AND “Neuroscience” = one result;
(5) “Serious Game” AND “EEG” AND “Learning” AND “Neuroscience” = two results;
(6) “Serious Game” AND “ET” AND “Learning” AND “Neuroscience” = one result.

Considering that the first selection resulted in only four articles, it was decided to remove the word “Neuroscience,” reducing the constraint, which considerably improved the results. Therefore, the searches were combined as follows:

(7) “Business Game” AND “EEG” AND “ET” AND “Learning” = three results;
(8) “Business Game” AND “EEG” AND “Learning” = 10 results;
(9) “Business Game” AND “ET” AND “Learning” = 13 results;
(10) “Serious Game” AND “EEG” AND “ET” AND “Learning” = 15 results;
(11) “Serious Game” AND “EEG” AND “Learning” = 90 results;
(12) “Serious Game” AND “ET” AND “Learning” = 86 results.

**Figure 1:**
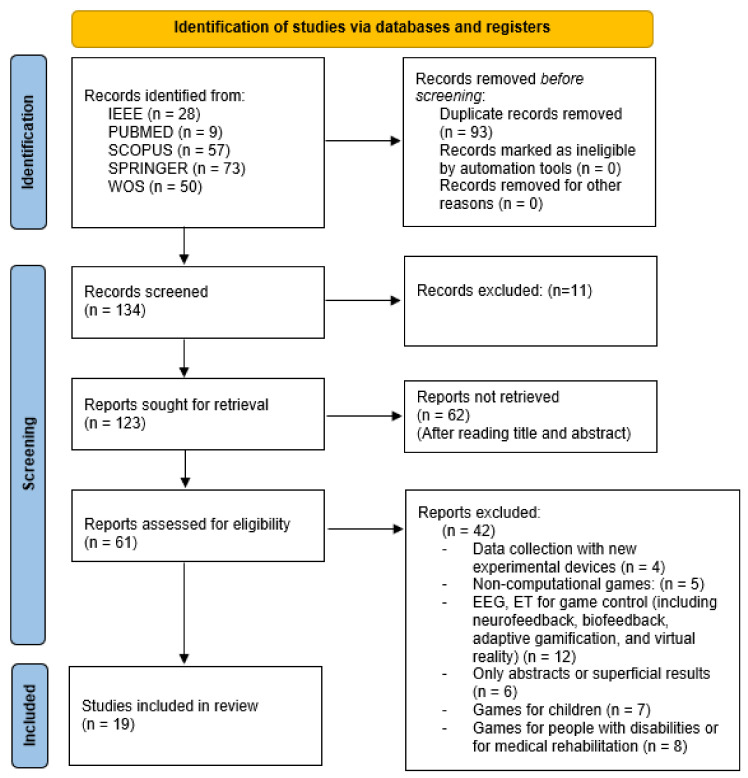
Visualization of the reviewing procedure using PRISMA 2020 flowchart.

Although the SR was designed to develop the theme with Business Game, as the result was only 26 studies, it was decided to also consider the word “Serious Game”, believing that it would be the most appropriate string, which provided more than 191 works. Thus, considering it satisfactory, the review work started from this set of 217 results (steps 7 to 12). The flowchart provides the number of studies obtained in the different databases in Figure 1.

### 3.2. Data Extraction

Potentially eligible articles were identified through a review of the article titles and abstracts in the database, which yielded 217 articles. After removing duplicates using the Mendeley tool, 134 articles were screened with deeper analysis of their abstracts resulting in 61 articles that required further assessment. After the complete reading of the studies resulting from the screening step, according to the PRISMA, another 42 documents were excluded from this SR for not falling within the scope defined initially, as detailed below:

- Data collection with new experimental devices (*n* = 4): researches contemplated equipment prototypes with unrecognized reliability;
- Non-computational games (*n* = 5): experiments with board games and puzzles;
- EEG, ET for game control (including neurofeedback, adaptive gamification and virtual reality (*n* = 12): investigations involving physiological devices with the purpose of direct control of the game and not as a tool for monitoring the user experience;
- Only abstract or superficial results (*n* = 6): studies without access to results or vague conclusions;
- Games for children (*n* = 7): investigations with user experience in games with an approach to literacy processes and preschool skills development;
- Games for people with disabilities or for medical rehabilitation (*n* = 8): research analyzing the use of serious games to enrich cognitive functions in people with a health deficit, which brings variables that are not comparable with monitoring of user experience with BSG.

Through full-text analysis, 19 fulfilled eligibility requirements. A visual description of this process, including the number of reports excluded separated by groups, is provided in Figure 1.

## 4. Results

This section presents an overview of the selected documents, seeks an initial approximation between them, and points out some important issues that culminated in this result. Table 1 was developed to facilitate the first look of the 19 publications finally selected. This table provides the initial characteristics, referring to the year of publication and the keywords met when included in this study.

Table 1 allows us to identify that 90% of the selected studies were published in the last 10 years, being only three involving BG [71,77,81], all of them from 2017, proving how few there are investigations on this topic. The reason decided to open the search for studies, including the keyword “SG.” Another important aspect is that few investigations were found addressing the two techniques EEG and ET, where their use is combined, drawing comparisons or bringing discussions about both [71,76,77,84], all of them in the last five years. It is important to note that, in this context, the word “learning” included in the search was essential to select only studies focusing on this topic. Thus, there are investigations into using these techniques in the health area, mainly in identifying and monitoring diseases that are not part of the scope of this research.

Table 2 was created to provide more specific information about each of the selected publications to discuss and interlocution results. This table briefly describes the objectives, methodology and main results, and conclusions. This table, however, also contributed to the identification of relevant issues to be considered in this section. One of them is that the selection resulted in five theoretical studies maintained as an object of investigation due to concepts and approaches to the questions proposed in this SR [72,76,77,86,87]. The contribution of the selected theoretical studies is crucial because it rescues elements that serve for an adequate contextualization of this SR, considering the importance of balancing theory and praxis to demonstrate and understand each step of a given phenomenon [89]. Another point identified as essential to highlight is that some of the selected articles present the analysis of games that at first glance appear to be exclusively for entertainment [79,81,82,88]. However, during their reading, it was possible to identify the methodology and analysis of the user experience supported by EEG or ET techniques, the contribution of skills and competencies while learning tool, assuming the role of a serious game and thus justifying inclusion study.

An investigation obtained from the search refers to the analysis of user experience based on EEG signals in a simulator for learning a medical procedure [80]. Considering that some types of BSG also have a profile more focused on equipment and process simulation, such as logistical or industrial operations, it was decided to include it in this SR and identify its possible contributions.

## 5. Discussion

In this section, we discuss the main findings of the studies summarized in Table 2. Also, it proposes implications for practice and deepening of the theme, based on the research question: What is the research related to the learning process with Business (Serious) Games using data collection techniques with Electroencephalogram Signals (EEG) or Eye tracking (ET)?

A study analyzing aspects of attention in a multiple-choice game addressing Chinese culture issues [70] introduces a novel ET measure of performance in social interaction serious games. The investigation also proves using this dispositive to monitor specific cognitive processes and collaborate with more consistent results to traditional measures of in-game performance, typically qualitative. Another significant contribution of this study concerns the relationship between previous knowledge of the theme developed and the final game score. That deserves to be tested with BSG considering that many company simulation environments use this storyline format.

An investigation developed a data collection model using an EEG device to investigate a player’s connection in a BSG using cognitive neuroscience methods [71]. It measured brain waves at frequencies of 0.5–30 Hz and working with four bands to identify the participant’s behaviors:

- Delta (0.5–4 Hz) for view empathy levels,
- Theta (4–8 Hz) for notice learning or remembering moments,
- Alpha (8–12 Hz) for detect moments of relaxation, it occurs when closing the eyes,
- Beta (12–30 Hz) is divided into low activity waves (12–15 Hz), medium waves (15–20 Hz) and high waves (18–30 Hz),

To associate the situations with an increase of energy, anxiety, performance, and concentration. A pre-survey was carried out to identify what type of games people like to play for fun and what elements of these games should be embedded to develop an economic game to encourage greater engagement, serving as a benchmark for the BSG development. Still, in the same study, another game already available on the market with the same theme will be tested to compare the engagement of the participants. The experiment proposal also includes the use of ET to identify what the participants pay particular attention to, corroborating the concern of using more than one data collection technique in a combined way. The procedure proposal is iterative, and it will be repeated until a satisfactory result is achieved, i.e., the appropriate degree of involvement of the gamer.

Using the EEG, the same brain wave classification and further aided by brain heat maps, besides complementary electrooculogram (EOG) and respiration techniques. During a serious game experience, a neurocognitive study examined how the psychophysiological indices related to arousal, attention, motivation, and vigilance can help clarify the players’ likelihood to learn declarative knowledge [73]. Was used Bloom Taxonomy to distinguish levels of difficulty and pre-and post-game answers to measure the value of demonstrated learning. Presenting a relation between fronto-hemispheric asymmetry and the attention level for learning, the results allowed us to infer that those with more balanced were more likely to score highly. These asymmetries identified are related to the distractors, even reported in the participants’ final questionnaire. It makes sense to monitor and consider them when developing a BSG, in line with design requirements already presented in this SR [42].

Also, within the scope of the analysis of the attentional state in SG, two studies included in this SR using the brain wave supported by EEG and complemented with other physiological devices, approximate results of user experience in strategy tasks with a theoretical model of motivation and the following components: Attention, Relevance, Confidence, and Satisfaction (ARCS). One of them was included the motivational measurement instrument called IMMS, used for each game mission to assess the element motivation and applying linear regression. Results presented that the theta wave in the frontal region and the high-beta wave in the left central region significantly predict users’ motivation [75]. The participants were separated into two groups in the other study to test Problem Solving and Alarm Trigger in two attention-getting strategies. The results point out that integrating results into an intelligent tutoring system can add the learner model to meet the real game design objectives [74].

Adopting the same ARCS motivation model adopted by two other studies included in this SR, an investigation includes collecting EEG data from a game developed specifically for the experiment. For the SG named HeapMotiv, four different strategies were implemented to analyze each element proposed by the theoretical model. The EEG signal was converted by Fast Fourier Transformation and divided into three bands:

- Theta (4–8 Hz),
- Alpha (8–13 Hz),
- Beta (13–22 Hz),
  obtaining the EEG mental engagement index [78]. The experiment also included a pre-test and a post-test. The result shows the relevant impact of motivational strategies on the level of involvement, reflected in the increase in the attention and vigilance rates of the students involved. The research concludes that the definition of motivational strategies is essential. Such strategies must adapt dynamically to the game and have an agent structure monitored and constantly evaluated using EEG devices.

The effect of different designs and end-users’ expertise on learners’ mental effort during gameplay is still an open question. For that purpose, a study investigates” How mental effort differs in the phases of the game (associated about) and relation to users’ expertise” using an EEG device [79]. This research also used brain wave monitoring, categorized into frequencies (alpha, beta, and theta), and estimated the involvement and effort in carrying out the task based on the amplitudes of the waves. The participants were college-level students, they knew the game, but none of them had played it at least two years ago. The experiment also used an EOG filter to remove blinking noise. The EEG signals were processed in MATLAB to extract the power spectral density (PSD). The PSD was extracted for the following bands for each of the four lobes (i.e., parietal, frontal, temporal, occipital). The results show that an elevation of the experience increases the levels of attention (alpha), allows the players to make decisions more easily (beta), and has a particular connection with aspects related to stress and memory (theta). However, it does not no direct relationship between experience and performance was identified. Based on the analysis of the EEG signals obtained, the study suggests that although several elements presented by the game may hinder a quick start, it is feasible to introduce them gradually in later stages of the game when the player is in a moment of greater concentration, more effective memory recovery, and faster decision making.

In a study exploring players’ performance in different levels of difficulty, a bronchoscopy simulator used questionnaires, game characteristic metrics, and EEG analysis from corresponding alpha, beta, delta, and theta brain waves and exported for spectral analysis performed using the MatLab software [80]. This study found that age influenced player performance, with younger participants demonstrated greater knowledge acquisition, completion assessment, and fewer errors. The EEG analysis revealed differing spectral profiles that may account for variations in player performance. Still, on this issue, the research identifies that while digital immigrants may have more difficulty learning through serious games, preliminary evidence suggests that SG does enhance training outcomes for digital natives. In terms of training regimes, more training sessions, rather than longer training sessions, would be most beneficial. The research concludes by suggesting the importance of identifying and understanding the different types of users of an SG and considering its design. These elements facilitate access and learning for digital immigrants.

Two investigations using ET analyze an SG in battle arena format with very different approaches. One of them [81] evaluates the ergonomics side of game related to the user interface design and monitor screen, evaluating task scenario that needs to be solved, including besides the ET, the Game Experience Questionnaire (GEQ), NASA TLX questionnaires and Questionnaire for User Interface Satisfaction (QUIS). The experiment compared the interface design and the type of monitor or screen size, measuring satisfaction, workload, previous experience, and game performance. The heat maps generated by ET, together with the other metrics adopted for performance evaluation, allowed us to conclude that if one of the game’s objectives is to have a shorter playing time, players will more easily complete these objectives if they use a larger screen (23 inches) screen with an interface design that is the size of an essential element larger than the standard size. Another essential bias identified in the research is that designs that establish information flow incompatible with the game flow will provide players with a considerably greater workload. This statement was proven from the analysis of the heat maps when verifying that the groove propagation in a 14-inch screen is clearer when compared to a 23-inch screen with a more divided appearance, which may be related to the capacity of the viewing angle. These findings warn how important it is to monitor elements related to the ergonomics of an SG and that the ET can offer significant support in this regard. The other investigation different evaluates how the same game type can foster hypothetico-deductive reasoning [82]. The methodology is based on multi-design and multi-level data triangulation between game videos, eye tracking, game talks, and cued retrospective protocols. With the study, still no results, the game recordings with eye tracking data will be in a simulated natural environment. Players can feel like playing a normal game at home while in a laboratory setting (i.e., wearing eye trackers). And intend to identify some trends for both the expert and the novice groups, using and developing cognitive skills to propose a new model of learning that can be used for BSG design.

Game-based learning was analyzed by ET technology to explore differences between (high and low conceptual players´ visual behaviors and game flow [83]. The experiment includes a pre-test to measure the theoretical concepts of the participants to divide the participants into two groups, and the SG screen was split into six Areas of Interest (AOI) to be analyzed by the ET device. From the analysis of heat maps, the findings suggest the importance of understanding and monitoring the users in conceptual understanding groups to propose elements for a game design that provides a better experience for all users. Another contribution of the ET measure is that the percentages of count and duration are a significant indicator for the flow experience in SG.

Employing an innovative methodology and protocol, a study articulates a systems dynamics model of learning based on a predictive cognitive architecture by interrelating six modules: external agents, knowledge, performance, affect, cognition, and context exploring the effect of agency on cognitive load from a group of dyads (one watcher and one player) playing an SG for learning physics, while dual-EEG (64 channels) was recorded for all participants [84]. As part of the more extensive study, ET, electrodermal activity (EDA), and blood pulse were also measured. However, these results are not shown in the paper and the Force Concept Inventory questionnaire filled pre-and post-test by the participants to measure their Physics knowledge. The partial results of the study point out that agency (the fact of being an active player or a passive watcher) has no systematic effect on the overall quantity of cognitive load. And because they are the same, individual differences from top to bottom suggest a more substantial impact on learning than the bottom-up effects of the learning context.

Another study that presents aspects of interest and motivation in a gamification experience developed an experiment analyzing two versions of the number line estimation task: one with game elements and one without [85]. During the test, participants completed both versions of the task while an ET recorded their eye fixation behavior, and the number and duration of fixations were recorded. The game’s display and the no-game condition were subdivided into different AOI to organize the data collected. Questionnaires were used to assess motivational aspects after each condition and categorize the Flow experience. The results showed differences in selective attention. It was possible to identify the eye fixation behavior, with no changes in performance between the game condition and no game, and a more significant experience of flow in tasks with game elements. In general, the results show that the ET contributes significantly to explore the users’ attention concerning the elements that a game presents, which would not have been identified so clearly if analyzed only from the conventional post-hoc questionnaires.

To analyze the player´s behavior in a cybersecurity game, a paper used EEG readings with a pre-and post-test of student knowledge and opinions regarding information security awareness and perceived immersion to help identify which readings would serve as the most indicative of a player’s perception of difficult [88]. The partial results of the investigation do not present the findings with the EEG device yet. However, the study’s goals include establishing a relationship between stress and attention that allows optimizing the SG resources so that its users remain in the flow state for a longer time, improving their experience and possibly learning results.

The theoretical studies included in this SR point out several contributions in studying BSG or SG related to EEG and ET. One of them is to establish insights of better using ET for SG to improve the learning experience. As a systematic literature review, the investigation highlights six further themes emerging from this analysis [72]. The study points out the importance of using ET to support in-game design, considering that this device can help identify learning models and individual user characteristics. Other benefits of tests with this device are related to better planning of the game control by the student (user), deliberate about the game information, and the importance of using it in conjunction with complementary user evaluation protocols.

Another investigation describes and discusses the measurement of evaluation constructs and operationalization as part of the SG design process [76]. The discussion is permeated by different methodologies and indicates physiological measurement, including EEG and ET, to assess indicators related to the players’ use of mental resources and attention. Guiding vantages and advantages, the study includes other measurement practices, including self-reports, observation, and gameplay metrics, without discarding any of them. And, on the contrary, recommending that more than one tool be used for a later cross-checking of data allows a more accurate result. Still, in collaborating with better evaluations in SG, an overview of taxonomies and questionnaires is presented. This can be applied depending on the concept sought to be evaluated, related to competencies and skills provided or no to the player by the game.

A state the art of BSG modeling supported by human-computer interfaces, more precisely in the use of ET and EEG, highlights that the studies already carried out in other segments can be transferred directly to business simulation environments [77]. Used alone or in combination, these devices unravel complex cognitive and behavioral mechanisms in the human experience with digital technologies. For example, the contributions from the EEG investigations, for example, for monitoring the learning effectiveness of medical procedure simulators and ET in controlling attention to advertising and creating referral systems, can help create BSG experimentation models, considering the quantitative few studies in the area.

Another theoretical approach establishes a conceptual adaptive data model dedicated to the SG. The model applies biomedical indicators and stimuli affecting the ability of learning and concentration, considering individual preferences and skills of the learner [86], using:

(1) ET to measure users’ concentration and dispersion of visual attention, scanning path, cognitive load;
(2) EEG to measure users’ concentration, engagement, fatigue, emotions, and cognitive load;
(3) Physiological equipment to analyze users’ behavior, for example, the level of stress.

The methodology proposal has a high dimensionality of variables. It allows a crossing of data from the various tools used in an integrated manner to provide valuable information in the development and implementation of SG.

A discussion about educational aspects and possibilities of SG describes key theories to ground the work of game designers through an inventory of known learning and affective outcomes in this type of game [87]. Presenting an analysis of the importance of relevant characteristics identified in recent studies on learning with games, the investigation does an overview of the following pertinent elements identified in studies on learning with games: learning styles, gender, age, and game literacy in the sense of predicting how learners will react on specific content, treatment and situations of the game. The crucial results show that the emotional state is monitored in-class observations, direct questionnaires, or that they are not always able to provide comprehensive information and, in large part, are not able to record the emotional experience. However, physiological or behavioral measures, such as ET or conductance of the skin, are shown as the most appropriate methods because they can be collected in real-time during the game. To ensure that a game is effective in helping those who learn to achieve the proposed goals, it is essential to consider how the learning content is incorporated into its elements and stages. In this perspective, this investigation contributes to affirm that physiological measuring devices are crucial elements in the design process of an SG, as well as the other theoretical studies selected and addressed in this SR.

Complementing the analysis of results, it should be noted that all the complete experiments considered in this SR used EEG devices of different brands and models. However, following the international standard of electrode disposition, and did not report any problems or difficulties regarding the measurement and accuracy of the results obtained. A factor that limited the level of interpretation of brain function in some studies was the number of electrodes used (which ranged from three to 64). About eye tracking, all experiments with ET used different models of the Tobii brand (glasses or headset), however, with the same near-infrared technology of glasses. No study has also reported problems or disadvantages in using the equipment.

With the consolidation of EEG and ET in the market, these technologies have been improving. Brain activity (EEG) sensors are often susceptible. All EEG experiments included in this investigation report modern filters to deal with noise and artifacts. The same is true for ET devices, which in turn offer eye shift correction tools. In the practical studies selected in this SR, the measures of continuous monitoring of the user’s mental state and fixations and saccades proved to be very relevant in identifying user satisfaction at each stage of the game, highlighting changes in the user’s behavioral state (such as lack of attention) and the occurrence of desired states (such as flow). The referred interfaces prove to be effectively important to propose elements for the game’s development, presentation, and difficulty levels with these tips.

Another noteworthy aspect that corroborates the fact that users did not report discomfort in the tests is that these technologies, although non-invasive, have been concerned with being increasingly “invisible,” allowing players to focus exclusively on the game. This does not rule out the concern of studies related to ergonomic aspects, also identified in this work.

## 6. Conclusions

This SR presents theoretical and practical contributions to implementing a structure to monitor user experience with BSG through ET and EEG devices. These measures were chosen because they are two recognized and accessible human-machine interfaces to identify design elements significant for skills and abilities. This investigation was able to bring together existing researches that contributes to the value of the user’s mental and visual information as a data source, during the skill acquisition phase when playing a SG, highlighting them as fundamental components for the definition of an interactive system. A major concern observed in the selected studies is related to the search for game elements that provide motivation and user engagement, through the identification of moments in which cognitive overload leads to performance failures and skills decline. In this sense, the theoretical contribution comes from the results of these investigations in which eye tracking and EEG measurements were successfully implemented to provide this assessment, allowing for more accurate feedback to act in the identification of errors and successes in the design of a SG, and which are perfectly applicable to BSG. This research looks at the existing scientific investigations that address concepts and practices using technological resources in the feedback on simulations and serious games, filling the current gap on quantitative studies with BSG, to go beyond results obtained from the point of view of your users. The practical contributions of this research take place from the moment that, by understanding the structures and methodologies already validated in equivalent studies with other learning tools, it is possible to replicate or adapt them to the BSG, reducing experimental stages. By adopting such physiological sensors that allow the identification of usability factors (positive and negative) in real-time and continuous gameplay, it will enable BSG designers to improve the quality of content quickly, without resorting to complex procedures and high user rating cost, which do not always provide clear and specific subsidies.

Literature data provide strong evidence for the role of SG analysis, including BSG, supported by human-computer interfaces, mainly ET and EEG, the focus of this study. All of the experiments and reviewed methodologies showed great promise and were capable of obtaining high-quality models of the user experience in this type of learning games. Multiple trends arise among the research. EEG experiences relating the results with the age of the participants contributed with reasonable accuracy to conclude that those who do not fit as digital natives have lower levels of cognition and memory and that this can make it challenging to understand the game and consequently reduce the expected level of learning. Already tests with ET show how the results for the duration and number of fixations can be excellent indicators of mental effort.

In the search for possible connections between the selected studies, what draws a lot of attention is the recurrent use of the term performance. By analyzing this common point, it is clear that it is a guide for investigations that establish relations between the measurements provided by sensors (TE or EEG) during the player’s achievement and his actual result in the game. At the same time that they prove the applicability of measuring devices in monitoring the experience of users with the SG, these researches point to contributions to the design of this learning tool. In each study, the adopted methodology seeks to prove, with the results obtained, aspects such as level of attention, mental effort, degree of stress, level of learning, perception of difficulty, flow, concentration, satisfaction, motivation, problem solving strategy and fatigue. In any aspect(s) addressed by the investigations, it is highlighted that the detection of signals occurs relatively simply and in real time, which significantly reduces the possibility of errors or inconsistent results. Data generation during the full game, made possible by these models of non-invasive data collection devices, is also a feature of all experimental investigations selected in this SR that should be adopted in experiments with BSG. Monitoring how the player behaves over time at different difficulty levels, phases, screens and state changes offered by the game provides comparable data that lead to very consistent conclusions. These results allow to effectively establish which game elements influence the acquisition and retention of knowledge and skills.

The main limitation of this work is the small amount of research relating ET and EEG devices to support the analysis of the BSG user experience, which is why the inclusion of studies was adapted to consider similar results obtained with serious games in general, seeking bring contributions closer together. One issue that may, at first glance, present itself as a restriction in the scope of the study is the fact that this research has centered its approach on the use of ET and EEG as a monitoring interface. In this aspect, it is important to rescue the background of this SR, which already points out these two techniques as the most recommended. Also, by going deeper into the reading of the 19 selected manuscripts, it is noticed in the investigations that include more than one interface in their methodology, that the ET and the EEG are always the main sources of data collection, and the other(s) device(s) play a complementary role. This perception is relevant to clarify that another sensors and technologies can contribute to this field, but it is also important to focus on techniques that effectively present more representative results. By rescuing these researches addressing sensing methodologies and technologies in the analysis of user experience, scientifically consolidated, it is expected to create a consistent reference to deepen studies with BSG.

If the ET and EEG techniques individually present relevant contributions to studies with BSG, using these physiological devices in an integrated manner, or any of the two with other equipment for collecting physiological signals, it can still offer even more quality to the researches in game design. The same is true for combining qualitative assessment methods, as pre-and post-tests, questionnaires to assess knowledge levels and psychological and emotional profile. That can contribute significantly to this interweaving of information from the moment that we understand the user better, what the user knows and perceives about issues of the game, the theme treated, emotional aspects, among others. Work with multiple results, quantitative and qualitative—either comparing them or combining them to develop a hybrid model, is an excellent alternative in the search for constructs that can assist in the design and improvement of SG.

Although EEG and ET techniques contribute effectively to the SG research process, knowing the different learning models, and defining which one to use, is a significant initial step to understand which user characteristics will be monitored and to define the correct and most suitable protocol of experimentation for the game in question. In general, understanding how the players’ cognition is processed and monitoring flow, attention, and motivation during different moments and phases of the game are shown as key elements in the investigations of this SR. Using all this knowledge, some of these studies show that previous experience and performance are unrelated, highlighting the importance of the tutorial or manual that levels the necessary knowledge for the tasks proposed by the game.

As future work, it is recommended to develop experimental researches monitoring the user experience specifically with BSG, through EEG and ET devices, aiming at contributions equivalent to those obtained in investigations with other types of games and simulators included in this SR. Obviously, a BSG provides design elements specific to the business theme that will need to be considered in elaborating an experimentation model and defining the highlights and elements of data collection. It is still important to emphasize that any investigation with BSG supported by physiological sensors, promoted here, should not forget to consider qualitative research on user perception, because as also highlighted in this work, behavioral tests before and after the experiment offer significant insights by crossing them with data obtained with ET, EEG and other complementary physiological devices, providing a very consistent source of information.

Another possibility arises for specific studies with EEG and ET in monitoring players’ emotional stimuli. Although some studies refer to the analysis of emotional traits of players in their experiences with the SG, all of them arise from psychological tests applied together in the experiment, confirming that effectively there is no quantitative research applied to the monitoring and evaluation of emotional aspects during the game. It is unanimous in the researches included in this SR that the degree of attention is an important information offered by the ET and EEG, but they do not explore the related emotions that act as internal signals in the mobilization of this cognitive resource. Bringing Neuroscience concepts closer to the data obtained with these devices can help, when using the ET, for example, to establish a relationship between the level of fixation on a specific point on the screen and emotions such as boredom, anxiety and excitement. In turn, monitor the activation of specific areas of the cortex with the EEG, relating it to the player’s satisfaction, joy and interest, can collaborate in the understanding of the actions and strategies adopted by him. Thus, it is believed that serious game designers could also benefit from this feedback.

Finally, rescuing the research question established as guiding this work, it is concluded that there is a research gap monitoring the experience of using BSG supported by EG and ET. However, the nineteen studies included in this SR, essentially involving SG, are an excellent approach so that protocols and methodologies for the analysis of interactive business simulation environments are also developed so that these tools are scientifically recognized as a learning resource and contribute to optimized design elements.

It is strongly believed that this study can motivate further research in this field. The evidence presented here shows that knowing and making the most of physiological devices and sensors is essential to discovering and improving digital learning tools, including applications in business environments.

## Figures and Tables

**Table 1:** List of studies included in the SR and the corresponding keywords served in the search.

Reference	Year	BG	SG	EEG	ET	LRN
[70]	2018		X		X	X
[71]	2020	X		X	X	X
[72]	2012		X		X	X
[73]	2014		X	X		X
[74]	2011		X	X		X
[75]	2010		X	X		X
[76]	2016		X	X	X	X
[77]	2020	X	X	X	X	X
[78]	2014		X	X		X
[79]	2019		X	X		X
[80]	2016		X	X		X
[81]	2017	X			X	X
[82]	2015		X		X	X
[83]	2016		X		X	X
[84]	2021		X	X	X	X
[85]	2020		X		X	X
[86]	2015		X	X		X
[87]	2016		X		X	X
[88]	2020		X	X		X

**Table 2:** Objectives, Methodology, and main results of the studies included in the SRL.

Ref	Objectives	Methodology/Technique	Main Results
[70]	Analyze in a social interaction SG: -Visual attention during dialogue proposed; -ET measure of performance correlates to other existing measures of performance.	-A game called CCDTS played by 25 participants tested individually through an executable Unity file. All users were initially submitted in a Cultural Intelligence—CQ—Inventory) and a training introducing the storyline. The ET recorded used Tobii Pro V1.1; -The fixation durations on specific areas of interest (AOI) were processed from the collected gaze data. All fixations were aggregated to determine the total fixation duration in selected responses.	-Participants generally spent more time fixating on specific choice than any other option and spent more time fixating on the correct alternative rather than incorrect options; -There is no direct relation between a high CQ score and a high game result; -Gamers who exhibited higher fixation duration percentages on their selected answers scored higher on the SG.
[71]	Develop a concept of a procedure for investigating the gamer’s involvement in a BSG using methods based on cognitive neuroscience.	-A proposal for an economic game to be played by 30 people while recording EEG signals to analyze specific game elements that cause low attention and engagement; -The experiment also includes ET to track what the tested person pays particular attention to and a pre-survey with the participants to develop the game.	Partial results point out: -A BSG about the financial market was elaborated from initial research, and a ready game existing in the market will be chosen to be used as a comparison; -Proposition of a measured model using the EEG signal to identify the player’s behavior while using the game.
[72]	Contribute towards a conceptual framework supported by ET for SG to improve user learning experiences.	-Systematic literature review analysis of the six identified articles rendered, using keywords ‘‘eye tracking AND serious games.”	-No conceptual framework is yet available for the overlapping area of ET evaluation of SG to improve user learning experiences; -ET provided thorough and objective information for SG interaction and design, which was helpful to developers during the development of storytelling, dialogue, and game structuring.
[73]	Presents a neuro-cognitive study of how users learn during an SG play, using EEG and the psychophysiology test of “learning game” players.	-45 participants were tested for topic comprehension by a questionnaire administered before and after playing the SG Peacemaker (Impact Games 2007), during the time which EEG and other physiological signals were measured.	-Multiple physiological dispositions support on-task behaviors and styles; -Dealing with a complex stimulus environment such as this SG, the most successful strategy seems to be one of balance between the brain’s hemispheres and between activation and dissociation.
[74]	Use of heart rate, skin conductance, and EEG and a theoretical model of motivation to evaluate two Attention-getting strategies in an SG.	-Using Keller’s ARCS model, 21 subjects played a game called Food Force. The participants were separated into two groups to test Problem Solving and Alarm Trigger in two Attention-getting strategies; -Sensors were attached to the fingers of participants’. EEG was recorded by using a cap with a linked-mastoid reference; -Three selected areas (C3, F3, and Pz) were placed according to the international 10-20 system; the reference and the ground sensors were located at Cz and Fpz, respectively.	-Some specific EEG ratios were more appropriate than others to evaluate learners’ behaviors and reactions physiologically; -Physiological evaluation of different attention-getting strategies can provide in a pertinent way an appropriate tool to discriminate between attentive and inattentive apprentices; The integration of these results in an intelligent tutoring system can contribute significantly to constructing the learning model for the student and adapting the tutor’s motivational interventions.
[75]	Investigate players’ motivation during an SG play, based on a theoretical model of motivation and from the collaboration of an EEG device.	-This study involved 33 participants, adopting the Keller’s ARCS Learning Motivational Model during the SG Food Force, based on a motivational measurement instrument called Instructional Materials Motivation Survey (IMMS); -Electrophysiological data were recorded during the whole of the experiment (galvanic skin response electrodes, the blood volume pulse sensor, and EEG signals).	-Multiple linear regression showed the statistical significance of specific EEG data; -Theta wave in the frontal regions and motivation were positively correlated; The high-beta wave in the left-center region was a relevant predictor for a high level of motivation, especially during the game’s final moments.
[76]	Collaborate with operationalization and measurement of evaluation constructs to be an essential step in the SG development process.	-As a theoretical study, first general data gathering methods are introduced to give an overview of available means to assess data, including ET and EEG; -Different constructs that are relevant in the context of SG evaluation are addressed to be able to derive concrete operationalizations; -For each construct, common ways to operationalize are presented, compared, and analyzed.	-Measuring physiological reactions or assessing in-game behavior of users´, researchers might also gain important insights by simply observing them; -Subjective data helps to find explanations and relations between observable behavior, physiological reactions, and processes; -Motivation is an essential part of the SG evaluation; hence, how this construct can be operationalized to measure it.
[77]	Presents the state of the art of BSG, with insights of Neuroscience, from the perspective of the user experience, through the support of EEG and ET devices	-Theoretical study integrating concepts and references related to Business Simulation Games, Neuroscience of Learning and Physiological and Neuroscientific Methods of Data Collection as support to analyze the user experience. -The article retrieves some crucial findings from existing research in the area and brings them closer to the BSG study.	-The use of BSGs for education as a learning resource was found that it has a qualitative bias; -The human-computer interfaces have not been used in studies to measure the experience in BSG. - Researches with simulators and games supported by EEG and ET interfaces have shown significant results to improve learning, including BSGs.
[78]	Evaluate the physiological responses of gamers´ strategies during their interaction with an SG, measuring the index of engagement using EEG.	-The study involved 20 participants adopting the Keller’s ARCS Learning Motivational Model during HeapMotiv gameplay; EEG data were recorded using the Emotive Epoc device during the experiment; -Pre-test and post-test to compare learners’ performance regarding the knowledge presented in the game.	-Motivational strategies have a positive impact in supporting overall motivation, engagement, and attaining high performance; -Agents provide an adaptable framework for SG design and respond quickly and autonomously to variable game situations according to the learner and trigger appropriate strategy.
[79]	Presents how mental effort differs in the phases of a game tested for adults (associated with the design) and users’ expertise using EEG data.	-Were captured the achieved score for each session of the game Pacman while collecting EEG data for each of the 17 participants and all sessions; -The participant had approximately 40 min to master the game and achieve a score that was as high as possible; -In particular, 20- channel EEG data were recorded following the international 10–20 system.	-Increasing game difficulty for more experienced players or adjusting the game as experience and performance increase could help players better utilize their cognitive abilities and enrich learning.
[80]	Explore players’ performance in a bronchoscopy SG simulator, using EEG, to analyze it as a consistent method for increasing skills and competencies.	-The study used questionnaires, gameplay characteristics, and EEG spectral (by Epoc Emotiv device) analysis to explore features of 15 players’ performance in a gamified simulation of a bronchoscopy; -Participants were divided into two groups of analysis based on the performance in the first session.	-Age influenced player performance, with youngers demonstrating greater knowledge acquisition through level progression and completion assessment; -As serious games provide a digital form of knowledge acquisition, in the ‘language’ of digital natives; -More training sessions, rather than longer training sessions, would be most beneficial.
[81]	Evaluate the ergonomics side of the game related to the user interface design and monitor screen through a case study on the game Battle Arena from the perspective of an SG.	-The research involved a total of 18 participants and include performance metrics, Game Experience Questionnaire (GEQ), NASA TLX questionnaires, Questionnaire for User Interface Satisfaction (QUIS), and ET (Tobiipro device), used in the field of cognitive ergonomics.	-If the desired in play is to have a shorter playing time, then players will more easily complete the game objectives if using a larger screen (23-inch screen) with interface design that has the size of an essential element larger; -If the desired play is related to perceived pleasure, players will prefer to play on a larger screen with larger information elements.
[82]	Investigate how commercial games can foster hypothetic-deductive reasoning in everyday life.	-Study of a Multiplayer Online Battle Arenas game while developing a design methodology based on multi-level data triangulation between game videos, eye tracking, and game conversations; - It establishes retrospective protocols with clues, analyzing the different reasoning processes of the players, addicted in an environment as natural as possible.	Partial results identify some trends: -Expert players make hypotheses, both in conscious and unconscious ways. Their hypotheses are related to opponent behavior, opponent intentions, opponent location, and the probability of the occurrence of a specific event; -Expert players try to acquire more information to test their hypotheses.
[83]	Using ET, explore differences between high and low conceptual comprehension players’ visual behaviors and game flow in an SG.	A total of 22 university students participated in this study. While playing a Physics game, their eye movements were recorded by an ET—The flow and comprehension test scores were collected.	-The players in the higher comprehension group demonstrated an efficient text-reading strategy and better metacognitive controls of visual attention during the game plays and expressed a higher level of game flow.
[84]	Explore effects of agency on cognitive load, articulating a systems dynamics model of learning based on cognition performance, knowledge, affect, external agents, and context.	-Thirty-six dyads played an SG Mecanika for learning physics while dual-EEG (64 channels per head plus reference and ground) was recorded. While one participant played, the other watched a real-time duplication of the player’s gameplay on a separate screen. A 20 min stop rule was established for every level to avoid discouragement caused by repetitive failure; -ET, electrodermal activity (EDA), and blood pulse were registered too, but not report.	-Time-series analysis shows that agency (player or watcher) does not affect the overall cognitive load when the comparison is made either by group or within a single dyad; -Nor did agency affect instantaneous cognitive load for a vast majority of dyads- as the learning environment does not produce even minimal correlations in cognitive load in both participants seeing the same thing.
[85]	Investigate the effect of game elements on behavioral performance, attention, and motivation using an ET device.	-Considering two versions of the number line estimation task—one with game elements (embedded in the SG called Semideus) and one without, 42 university students completed both versions of the task (to locate fractions on a number line ranging from 0 to 1). At the same time, their eye fixation behavior was recorded using a Tobii 1750 ET (Tobii Technology).	-Participants paid attention to game elements, although they were not necessarily completed the task; -The game elements seem to capture attention but also increment motivational aspects of learning tasks rather than decreasing performance; -The observed qualitative differences in fixation behavior might also originate from an increased user and flow experience.
[86]	Presents a conceptual adaptive data model of SG design complying with individual preferences and abilities to enhancing the learning process.	-The theoretical study describes applied methods relating Bayesian networks, EEG data processing, Emotion recognition, and classification based on EEG, ET, and other biomedical signals and presents an adaptive data model.	The data model includes the integrated biomedical data: -EEG data filtered and processed to determine user’s state of being; -ET data processed to determine user points of interest, fixations, and points of focus as well as the pupil size indicating the excitation/ concentration; -EEG signals and ET data (pupil size, fixation rate) need to be integrated to confirm the user’s state of being. Through them, emotions such as irritability, relaxation, nervousness, and excitement might be detected.
[87]	Discuss educational aspects and possibilities of SG, describing key learning theories to ground researchers and game designers’ work.	The theoretical study draws meta-reviews to offer an inventory of known learning and affective outcomes in serious games and to discuss assessment methods valuable for research and efficient SG design, including analysis using EEG; - Different individual characteristics that seem to be strongly affecting the process of learning with SG (learning style, gender, and age) are discussed with emphasis on game development.	-Emotional state is mainly monitored within class observations or questionnaires that not always provide comprehensive data and cannot broadly capture emotional behavior; -For Physiological or behavioral measures, the ET is one of the most appropriate methods because it can be collected during gameplay; Different people learn and process information differently; it is essential to understand how learners react to specific content and situations.
[88]	Describes a study of player behavior and EEG readings while playing the cybersecurity SG Brute Force, a tower defense game that teaches players to choose passwords.	The experiment with 28 participants included: -pre-test regarding their knowledge and attitudes toward passwords; -a brief tutorial and game playing with linear progressive difficulty adjustment for up to 15 min, using the EEG headset (Emotive device); -a post-test to measure any changes from the pre-test scores, along with a questionnaire regarding player experience.	Partial results show: - Player password selection (that is, choosing a password from a list) sometimes improves with more extended playtime; -The EEG normalized stress graphic shows that while the amplitude seems less informative, the frequency of spikes appears to be pretty well correlated to the perception of difficulty.

## Data Availability

Not applicable.
